# The Role of Semaphorin 3A in Bone Remodeling

**DOI:** 10.3389/fncel.2017.00040

**Published:** 2017-02-28

**Authors:** Zhenxia Li, Jin Hao, Xin Duan, Nan Wu, Zongke Zhou, Fan Yang, Juan Li, Zhihe Zhao, Shishu Huang

**Affiliations:** ^1^State Key Laboratory of Oral Diseases, National Clinical Research Center for Oral Diseases, West China Hospital of Stomatology, Sichuan UniversityChengdu, China; ^2^Program in Biological Sciences in Dental Medicine, Harvard School of Dental MedicineBoston, MA, USA; ^3^Department of Orthopaedic Surgery, West China Hospital, Sichuan UniversityChengdu, China; ^4^Department of Orthopaedic Surgery, Peking Union Medical College Hospital, Peking Union Medical College and Chinese Academy of Medical SciencesBeijing, China; ^5^The Brain Cognition and Brain Disease Institute, Shenzhen Institutes of Advanced Technology, Chinese Academy of SciencesShenzhen, China

**Keywords:** Semaphorin 3A, skeleton, remodeling, osteoprotection, nerve innervation

## Abstract

Bone remodeling occurs at the bone surface throughout adult life and associates bony quantity and quality. This process is a balance between the osteoblastic bone formation and osteoclastic bone resorption, which cross-talks together. Semaphorin 3A is a membrane-associated secreted protein and regarded as a diffusible axonal chemorepellent, which has been identified in the involvement of bone resorption and formation synchronously. However, the role of Semaphorin 3A in bone homeostasis and diseases remains elusive, in particular the association to osteoblasts and osteoclasts. In this review article, we summarize recent progress of Semaphorin 3A in the bone mass, homeostasis, and diseases and discuss the novel application of nerve-based bone regeneration. This will facilitate the understanding of Semaphorin 3A in skeletal biology and shed new light on the modulation and potential treatment in the bone disorders.

## Introduction

Semaphorin 3A (Sema3A), also known as C-Collapsin-1, H-Sema III, M-SemD, R-Sema III, Sema-Z1a, is a membrane-associated secreted protein (Behar et al., 1996; Ieda et al., 2007; Fukuda et al., 2013). It was originally identified as a diffusible axonal chemorepellent that modulates axon guidance and growth. Sema3A belongs to the semaphorin family and is firstly identified in the involvement of patterning neuronal connections. This includes neuronal migration and axon guidance in the development of the nervous system (Zanata et al., 2002; Kruger et al., 2005). Semaphorin protein was firstly found in brain that induces the collapse and stalling of neuronal growth cones, which was named collapsin (Luo et al., 1993). Up to date, semaphorin family has been regarded as one of the key chemorepellents or chemoattractants that is involved in axon steering, fasciculation, branching and synapse formation (Goodman et al., 1999). In particular, Sema3A is not only a chemorepellent for cortical axons but also a chemoattractant for cortical apical dendrites (Polleux et al., 2000). Although Sema3A was initially identified in the neuronal guidance, it has further been reported in the association of bone biology, cardiac development, cancer progression and immune disorders (Behar et al., 1996; Moretti et al., 2008; Carrer et al., 2012). These findings suggest that Sema3A is involved in the organogenesis, vascularization and angiogenesis (Behar et al., 1996; Neufeld and Kessler, 2008; McKenna et al., 2014).

Central and peripheral nerve systems play important roles in the regulation of bone which is a densely innervated structure (Calvo and Forteza-Vila, 1969; Serre et al., 1999). This process includes many factors such as leptin, neuropeptide Y (NPY), cocaine and amphetamine regulated transcript (CART) and neuromedin U (NMU; Takeda, 2008). Sema3A has been reported to repel trigeminal and dorsal root ganglia which are involved in bone innervation (Kitsukawa et al., 1997; Taniguchi et al., 1997). Recent studies have suggested that Sema3A can suppress bone resorption and enhance bone formation synchronously (Hayashi et al., 2012; Negishi-Koga and Takayanagi, 2012). However, the role of Sema3A in the osteogenesis and osteclastgenesis still remains elusive.

In this review article, we will discuss the recent progresses of Sema3A in the bone mass, homeostasis and diseases and highlight the novel findings of Sema3A in the regulation of osteoblasts and osteoclasts. Additionally, this will facilitate the understanding of its role in skeletal biology and shed new light on the modulation and treatment in the bone disorders.

## Semaphorin 3A and Its Receptors

**Figure 1:**
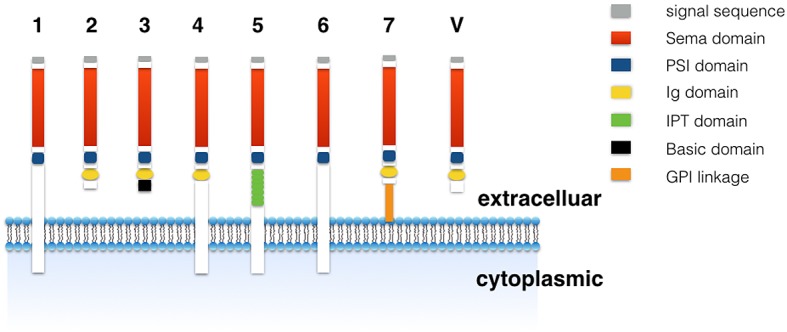
**The subunits of semaphorin family.** The semaphorin family has been categorized into eight classes. The class 1 and 2 are present in the invertebrate, whereas class 3–7 are present in vertebrates. The class V are encoded by viruses.

The semaphorin family has a conserved 500 amino-terminal seven-bladed β-propeller semaphorin domain. A short cysteine-rich plexin-semaphorins-integrin (PSI domain) is in the N-terminal and additional sequence motifs are in the class-specific C terminus (Goodman et al., 1999; Janssen et al., 2010; Nogi et al., 2010). The semaphorin family includes eight subclasses (Figure 1). The class 1 and 2 are present in the invertebrate, whereas class 3–7 are present in vertebrates (Goodman et al., 1999). The class V is encoded by viruses. These classes differ in membrane anchorage that includes secreted, transmembrane (TM), and glycosylphosphatidylinositol (GPI)-linked types (Goodman et al., 1999). TM domain exists in class 1, 4, 5 and 6 while GPI linkage only exists in class 7 (Goodman et al., 1999). Immunoglobulin (Ig) domain is present in all classes except class 1, 5 and 6. The class 3, one of the only secreted forms of semaphorin in vertebrates, contains semaphorin domain, Ig domain and short basic domain (Goodman et al., 1999).

**Figure 2:**
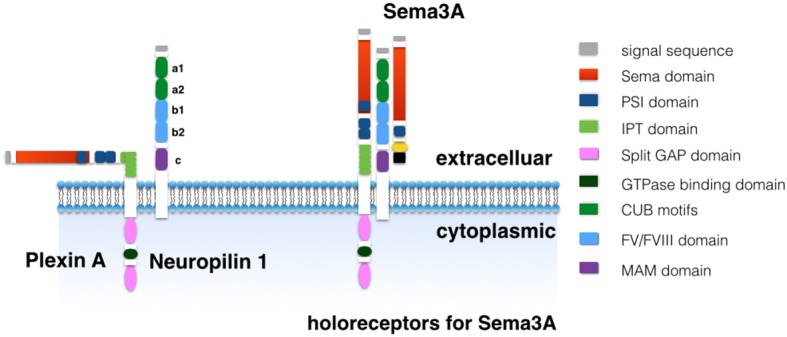
**Holoeceptors for semaphorins.** Plexin A has an N-terminal sema domain followed by three PSI domains plus six Ig domain shared by plexins and transcription factors (IPT) domains in extracelluar. The intracellular domain comprises two split cytoplasmic GTPase-activating protein (GAP) domains and a GTPase binding domain. The neuropilin1 has a large extracellular domain, a single transmembrane domain, and a short cytoplasmic tail. The extracellular domain comprises two N-terminal CUB motifs (domain a1 and a2), two coagulation factor V/VIII homology domains (domain b1 and b2) and a membrane-proximal MAM domain (domain c).

Plexins and neuropilins family are two major receptors for semaphorins (Figure 2). Plexins are one of the large type 1 single-transmembrane-spanning cell surface receptors which comprise at least nine members in four subclasses, plexin A, B, C and D (Janssen et al., 2010). Plexins show high structural homology and share the same signature feature with semaphorins. This is an N-terminal semaphorin domain followed by a short PSI domain. Furthermore, plexins contain two additional PSI domains plus six Ig domains shared by plexins and transcription factors (IPT; Janssen et al., 2010; Nogi et al., 2010). Plexins have been involved in the binding of semaphorins that play a role in the signal transduction. For example, Plexin B1 is a receptor for semaphorin 4D and plexin C1 is a receptor for semaphorin 7A, and plexin As (PlexinA1, A2, A3 and A4) are receptors for the class 3 and 6 semaphorins (Kolodkin et al., 1997; Takahashi et al., 1999; Tamagnone et al., 1999; Tran et al., 2007). Plexin A can directly bind semaphorin 6 through contacts of the conserved part of the semaphorin domains (Janssen et al., 2010). However, plexin A binds class 3 semaphorins in an indirect way, as plexin A conjugates one of two neuropilins (Neuropilin 1 or Neuropilin 2) to generate holoreceptors for these semaphorins (Kolodkin et al., 1997; Takahashi et al., 1999; Tamagnone et al., 1999; Tran et al., 2007). Additionally, neuropilin 1 (Nrp1) and neuropilin 2 (Nrp2) bind to Sema3A with different affinities (He and Tessier-Lavigne, 1997; Kolodkin et al., 1997). One of the major determinants for the affinity is the C terminus of semaphorins (Giger et al., 1998; Lee et al., 2003). For example, semaphorin 3F binds to Nrp2 with high affinity and to Nrp1 with much lower affinity. Conversely, Sema3A binds to Nrp1 with high affinity and this is an exclusive signaling pathway (He and Tessier-Lavigne, 1997; Kolodkin et al., 1997).

The neuropilins are single-transmembrane-spanning cell-surface glycoproteins that have a large extracellular domain, a single transmembrane domain, and a short cytoplasmic tail. The extracellular domain comprises two N-terminal CUB motifs (domain a1 and a2), two coagulation factor V/VIII homology domains (domain b1 and b2) and a membrane-proximal MAM domain (domain c; Soker et al., 1998; Antipenko et al., 2003; Janssen et al., 2012). Both domain a and b are essential in the binding of the Sema3A. Domain b1 is involved in the interaction with vascular endothelial growth factor (VEGF) and the basic carboxy terminus of Sema3A. Domain c has been reported to mediate neuropilins dimerization that interacted with other membrane receptors. Domain c indirectly interacts with either semaphorin or VEGF ligands. For example, Sema3A need co-receptors formed by neuropilins and plexin As (Chen et al., 1998; Giger et al., 1998; Nakamura et al., 1998; Lee et al., 2003).

## Sema3A Emerges in Skeletal System

Bone tissue is renewed throughout adult life and this process is called bone remodeling (Negishi-Koga and Takayanagi, 2012). It mainly occurs at the bone surface and includes the osteoblastic bone formation and osteoclastic bone resorption. This remodeling is a balance not only for normal bone mass and strength, but also for mineral homeostasis. If the balance has been disrupted between the bone formation and resorption, it causes bone disorders including osteoporosis, osteopetrosis, osteonecrosis (Teitelbaum, 2000; Grayson et al., 2015). Bone remodeling not only relies on bone cells including osteoblasts, osteoclasts and osteocytes, but also is linked tightly through coupling factors, including growth factors released from the matrix, soluble and membrane products of osteoclasts and their precursors, signals from osteocytes and from immune cells and signaling taking place within the osteoblast lineage (Negishi-Koga and Takayanagi, 2012).

Numerous factors have been identified in the participation of bone remodeling. Receptor activator of nuclear factor κB (RANK), receptor activator of nuclear factor κB ligand (RANKL), macrophage colony-stimulating factor (M-CSF), Osteoprotegerin (OPG), interleukin (IL) and many other factors have been demonstrated to associate with bone resorption. Transforming growth factor β (TGF-β), bone morphogenetic proteins (BMPs), Wnt signaling have been shown to be involved in bone formation (Sims and Martin, 2014). Most of these factors participate in the regulation of one aspect, either bone formation or resorption. For example, OPG protects bone by inhibiting osteoclastic bone resorption rather than the stimulation of osteoblastic bone formation (Togari et al., 2000).

Up to date, studies have suggested that Sema3A is in the regulation of bone resorption and formation synchronously. It suppressed bone resorption and increased bone formation to exert an osteoprotective effect (Hayashi et al., 2012; Negishi-Koga and Takayanagi, 2012). Behar et al. (1996) have generated *Sema3a* deficient mice by homologous recombination. Although the homozygous progeny were similar to their littermates at birth, most of them died over the following 3 months, only 12% were viable to adulthood. The surviving homozygous mice not only showed a paucity of neuropil, abnormal neuron orientation in the cerebral cortex, inappropriate sensory axons projection into the spinal cord, but also showed hypertrophy of the right ventricle, dilation of the right atrium and certain embryonic bones and abnormal development in cartilage (Behar et al., 1996). Knockout of the Sema3A gene also induced abnormal bone and cartilage development of the rat (Gomez et al., 2005). Additionally, Sema3A has been reported to precede or coincide with the invasion of bone by blood vessels and nerve fibers, not only in the temporal but also at the spatial level. Sema3A and its receptors were identified in the prehypertrophic and hypertrophic chondrocytes in ossification centers, around the periosteum, with the onset of endochondral ossification and vascular invasion (Gomez et al., 2005). Taken together, Sema3A is expressed in bone and involved in the regulation of innervation and blood vessel invasion, which may contribute to skeletal patterning.

## Sema3A Affects Osteoblasts and Osteoclasts

Sema3A signaling system is generally expressed in chondrocytes, osteoblasts and osteoclasts (Behar et al., 1996). Sema3A is mainly expressed by osteoblasts and its receptor Nrp1 is expressed by osteoclast precursors. Osteoclastic-like multinucleated cells from bone marrow have been demonstrated to express the ligands and receptors of Sema3A already (Koshihara et al., 1999; Togari et al., 2000).

Hayashi et al. (2012) observed a severe osteopenic phenotype in *Sema3a* knock-out mice (*Sema3a*^−/−^ mice), which was caused by a decrease in the osteoblastic bone formation and an increase in osteoclastic bone resorption. Fukuda et al. (2013) found that *Sema3a*^−/−^ mice showed a 25% decrease in the bone mass due to a decrease in bone formation without an overt change in the bone resorption at 3, 6 and 12 months of age, although a non-significant increase was observed in a serum bone resorption marker. *In vitro*, *Sema3a*^−/−^ osteoblasts showed a defect in cellular differentiation, with a decrease in alkaline phosphatase (ALP) activity and osteoblast markers, although the Sema3A treatment has rescued the defects in osteoblast differentiation. By contrast, the Sema3A treatment caused a decrease in osteoclast differentiation of osteoclast precursors, which the multinucleated TRAP-positive osteoclasts and the expression of osteoclast marker genes decreased (Fukuda et al., 2013). As such, Sema3A is regarded to increase bone mass in an autocrine fashion that stimulate osteoblast differentiation and inhibit osteoclast differentiation.

**Figure 3:**
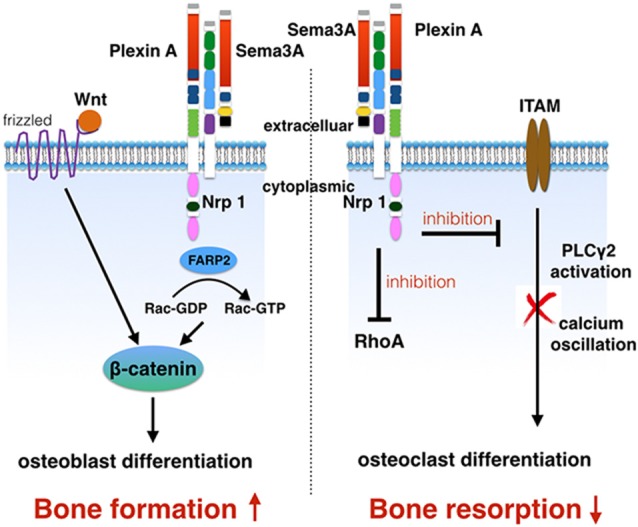
**Sema3A signaling in bone remodeling.** Sema3A increases bone mass by the stimulation of osteoblast differentiation and inhibition of osteoclast differentiation. Same3A regulates osteoblasts through Wnt/β-catenin pathway. Sema3A inhibits osteoclast differentiation through the arresting of PLCγ activation and calcium oscillation. Additionally, the inhibition of RhoA suppresses the migration of osteoclast precursors that are bone marrow-derived monocyte/macrophage precursor cells (BMMs).

For the pathway of Sema3A regulated-osteoblasts, Wnt pathway has been identified already (Hayashi et al., 2012). *Sema3a*^−/−^ mice and *Nrp1*^Sema−^ mice (mutant Nrp1 lacking the Sema binding site) showed a decreased osteoblast number, a reduced bone formation rate, and an increased adipocyte number. Calvarial cells obtained from *Sema3a*^−/−^ and *Nrp1*^Sema−^ mice were cultured in an osteogenic medium with or without Sema3A. ALP activity and bone nodule formation were markedly decreased and the adipogenic differentiation was highly increased in both *Sema3a*^−/−^ and *Nrp1*^Sema−^cells. This has been rescued by the treatment of Sema3A in *Sema3a*^−/−^ cells rather than in *Nrp1*^Sema−^cells. In *Sema3a*^−/−^ cells, the expression of Runx2, Sp7, Alpl and Bglap genes, associated with osteoblasts, were strongly suppressed, and the expression of Pparg, Cebpa, Fabp4 and Lpl, associated with adipocytes, was highly increased. It is believed that Sema3A activated osteoblast differentiation and inhibited adipocyte differentiation through Nrp1 (Hayashi et al., 2012). The canonical Wnt/β-catenin signaling pathway played an important role in the promotion of the osteogenic differentiation of MSCs. β-catenin has been identified to induce osteogenic differentiation and inhibit adipogenic differentiation (Case and Rubin, 2010; Cawthorn et al., 2012). Both the mRNA expression of most of the transcriptional targets of β-catenin and the Wnt3a-induced nuclear accumulation of β-catenin were suppressed in *Sema3a*^−/−^ calvarial cells (Hayashi et al., 2012). Rac1 activation can promote nuclear accumulation of β-catenin in response to Wnt signaling (Wu et al., 2008). Notably, in *Sema3a*^−/−^ calvarial cells, the activation of Rac in response to Wnt3a treatment was significantly decreased. Sema3A facilitated the nuclear translocation of β-catenin and the activation of Rac (Hayashi et al., 2012). Sema3A stimulates Rac1 activation through FARP2, which is a Dbl-family guanine nucleotide exchange factor (GEF) involved in the response of neuronal growth cones (Toyofuku et al., 2005; He et al., 2013). As such, it is regarded that Sema3A activates the canonical Wnt/β-catenin pathway in the process of osteoblast differentiation. FARP2-mediated activation of Rac1 was involved in this response (Hayashi et al., 2012; Figure 3).

Sema3A has been reported in the suppression of osteoclast differentiation. Osteoblasts express two cytokines, M-CSF and receptor activator of nuclear factor-κB ligand (RANKL), which are essential for the osteoclast differentiation (Boyle et al., 2003; Yamashita et al., 2012). Bone marrow-derived monocyte/macrophage precursor cells (BMMs) are osteoclast precursors. BMMs expressed c-Fms (M-CSF receptor) and RANK (RANKL receptor) and differentiated into osteoclasts in the presence of M-CSF and RANKL (Yamashita et al., 2012). OPG produced by osteoblasts was a soluble decoy receptor for RANKL and inhibited osteoclastogenesis by blocking the RANKL-RANK interaction (Simonet et al., 1997; Boyle et al., 2003).

Sema3A inhibited M-CSF-induced osteoclast differentiation through Rho A signaling pathway (Hayashi et al., 2012). A repulsive effect of Sema3A has been identified on the M-CSF-induced migration of BMMs (Hayashi et al., 2012). It is known that M-CSF induced the activation of the RhoA and Rac GTPases. Sema3A treatment abrogated the activation of RhoA activation in response to M-CSF, rather than the Rac activation. Additionally, the inhibition of RhoA activation caused the inhibitory effect of Sema3A on the migration of BMMs (Hayashi et al., 2012). Sema3A inhibited RANKL-induced tyrosine phosphorylation of phospholipase Cγ2 (PLCγ2) and calcium oscillation through immune-receptor tyrosine-based activation motif (ITAM) signaling pathway (Hayashi et al., 2012). RANKL-induced osteoclastogenesis was dependent on a co-stimulatory receptor signaling through ITAMs, including Fc receptor common γ (FcRγ) and DNAX-activating protein of 12 kDa (DAP12; Koga et al., 2004). RANK and ITAM signaling cooperated to induce NFATc1, which is the transcription of osteoclast-specific genes. FcRγ and DAP12 were associated with osteoclast-associated receptor (OSCAR) and triggering receptor expressed on myeloid cells 2 (TREM2; Yamashita et al., 2012).

PlexinA1 has been reported to interact with TREM2 and DAP12 to form the receptor complex for Sema6D. This ligand/receptor complex stimulated osteoclast differentiation in osteoclast precursors by the ITAM signaling pathway (Takegahara et al., 2006). Usually, Nrp1 as a receptor of Sema3A forms a receptor complex with PlexinA1 in BMMs (Takahashi and Strittmatter, 2001). With the increasing expression of Nrp1, the amount of PlexinA1 binding to Nrp1 increased and PlexinA1 binding to TREM2 decreased. With the down regulation of Nrp1, RANKL induced the formation of the PlexinA1-TREM2-DAP12 complex to release PlexinA1 from the PlexinA1-Nrp1 complex. The Sema3A treatment inhibited RANKL-induced formation of the PlexinA1-TREM2-DAP12 complex by the suppression of Nrp1 and the maintenance of the PlxnA1-Nrp1 complex (Hayashi et al., 2012). As such, the Sema3A-Nrp1 axis inhibited the osteoclast differentiation, which separated the PlexinA1 from the PlexinA1-TREM2-DAP12 complex. This further suppressed the ITAM signaling (Figure 3).

## Sema3A Regulates Bone Remodeling Through Sensory Nerves

It has been generally regarded that bone remodeling is mainly dependent on the local environment that includes autocrine and paracrine mechanisms. With the new discovery of leptin in the regulation of bone mass, both central and peripheral nervous systems has been noted in the study of bone development and homeostasis (Sandhu et al., 1987; Hill et al., 1991; Edoff et al., 1997; Takeda et al., 2002; Elefteriou et al., 2014). However, the molecular mechanisms by which neurons reach their targets in bones are not very clear. Several families of proteins involved in wiring and allow neurons to locate and reach their targets, such as Semaphorins, Netrins, Slits and Ephrins (Gomez et al., 2005). Togari et al has demonstrated the constitutive expression of neorotrophins, Sema3A, netrin-1 and netrin-2-like protein in human osteoblastic and osteoclastic cells. These axon guidance molecules are known to function as a chemoattractant and/or chemorepellent for growing nerve fibers (Togari et al., 2000). Particularly, it has been reported that Sema3A signaling is very important for neuronal targeting in the peripheral nervous system (Janssen et al., 2010; Nogi et al., 2010).

It has been reported that Sema3A works as an autocrine factor for neuronal development and neuron-derived Sema3A contributes to normal nervous system development (Fukuda et al., 2013). For instance, neuron-specific Sema3A deficient mice, like *Sema3a^−/−^* mice, showed abnormal development in the olfactory system and spinal cord, decreased thickness of the cerebral cortex, and altered pattern of sympathetic innervation in the heart (Behar et al., 1996; Ieda et al., 2007; Fukuda et al., 2013). Neuron-specific Sema3A deficient mice were generated based on synapsin-I-Cre mice (*Sema3a*_synapisin_^−/−^) and nestin-Cre mice (*Sema3a*_nestin_^−/−^; Zhu et al., 2001; Okada et al., 2006). Neuron-specific Sema3A deficient mice, like *Sema3a*^−/−^ mice, had a phenotype of a low bone mass, which was due to the decreased bone formation and increased bone resorption (Fukuda et al., 2013). However, the osteoblast-specific Sema3A deficient mice have been identified in normal bone formation and bone mass (Rodda and McMahon, 2006). As such, neurons in the bones were believed to take the responsibility for a substantial amount of Sema3A expression and the decreased expression of *Sema3a* in bone was only one of the causes of bone abnormality (Fukuda et al., 2013).

**Figure 4:**
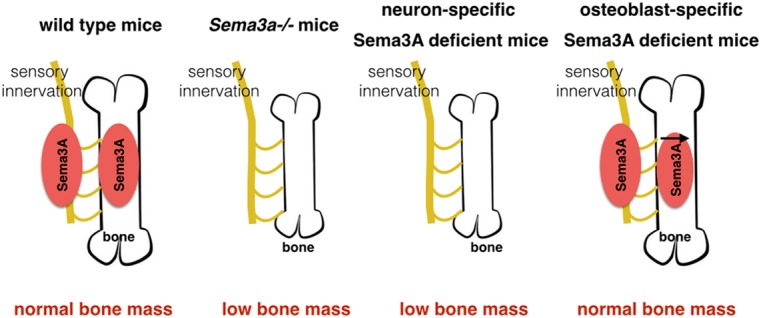
**Sema3A regulates bone remodeling through sensory innervation.** Neuron-specific Sema3A deficient mice, like *Sema3a*^−/−^ mice, have low bone mass. Osteoblast-specific Sema3A deficient mice have normal bone formation and bone mass, although the expression of Sema3A decreases in bone. Additionally, neurons in the bones are regarded to play a major role in the expression of Sema3A.

Neurofilament-positive fibers, involved in peripheral nerve innervations, were significantly decreased in both Sema3a^−/−^mice and neuron-specific Sema3A deficient mice (Fukuda et al., 2013). Additionally, sensory-positive nerves markers such as CGRP and TRPV1 were decreased in bone of *Sema3a*^−/−^ mice and neuron-specific Sema3A deficient mice. However, dopamine β-hydroxylase (DBH)-positive sympathetic nerve fibers, inhibited bone mass accrual (Takeda et al., 2002), were not significantly affected in these two mice models (Fukuda et al., 2013). The decreased sensory innervations are consistent with decreased bone mass in neuron-specific Sema3A deficient mice. By contrast, in osteoblast-specific Sema3A-deficient mice, which had normal bone mass but decreased Sema3A expression in bone, the projections of peripheral nerve fibers into bone tissues were not affected. As such, the abnormal projections of sensory fibers, not the expression of Sema3A in bone, are responsible for the bone abnormalities in *Sema3a*^−/−^ and neuron-specific Sema3A deficient mice (Fukuda et al., 2013; Figure 4).

Fukuda et al. (2013) were the first to demonstrate that sensory nerves play an important role in bone remodeling. Recently, some interactions between sensory neurons and sprouting sympathetic fibers have been observed (Xie et al., 2011). The sympathetic nervous system is known to inhibit bone mass accrual. Therefore, an interesting hypothesis is that there may exist a balance between “osteo-anabolic” afferent sensory nerves and “osteo-catabolic” efferent sympathetic nerves (Fukuda et al., 2013).

Recent evidence suggests that both central and peripheral nervous systems regulate bone remodeling (Karsenty and Ferron, 2012). Based on a number of anatomic, pharmacologic and genetic studies, the sympathetic nervous system represents one of the main links between the central nervous system and the skeleton (Elefteriou et al., 2014). More evidence is needed to demonstrate whether Sema3A regulates bone remodeling through the central nervous system.

## Potential Targets for Bone Diseases

To determine the therapeutic potential of Sema3A, Hayashi et al injected wild-type mice with recombinant human Sema3A. Intravenous administration in 5-week-old male mice led to increased trabecular bone volume and trabecular parameters in the distal femur. Bone morphometric analysis showed a decrease in osteoclastic parameters and an increase in osteoblastic parameters synchronously. Intravenous administration of Sema3A also accelerated bone regeneration in a mice model of bone cortical bone defects. As Sema3A treated mice showed higher regenerated cortical bone volume and significantly increased osteoblast surface and decreased osteoclast surface around the injured region. Additionally, Sema3A administration rescued bone loss in an ovariectomized mouse model of postmenopausal osteoporosis. *In vitro* experiment is consistent with *in vivo* as Sema3A suppressed osteoclastogenesis and promoted osteoblastogenesis in cultured human cells (Hayashi et al., 2012).

Local injection of Sema3A into the injury site in osteoporotic rats increased callus volume and density at 4 weeks post-fracture, and induced promoted callus ossification and remodeling at 8 weeks post-fracture (Li et al., 2015). Furthermore, rheumatoid arthritis synovial tissues showed decreased Sema3A expression (Takagawa et al., 2013). Sema3A is overexpressed in osteoarthritis cartilage and could play a role in chondrocyte cloning through inhibition of cell migration in osteoarthritic cartilage (Okubo et al., 2011). Sema3A is also proposed as a marker for systemic lupus erythematosus (Vadasz et al., 2012). Similarly, Sema3A may be a marker for bone disorders such as osteoporosis. Sema3A not only repels neuronal ingrowth but also may repel neovascularization within the healthy disc, this makes Sema3A as a potential therapeutic target in the treatment of low back pain (Tolofari et al., 2010).

In conclusion, Sema3A exerts an osteoprotective effect by both suppressing bone resorption and increasing bone formation at the same time. Sema3A can regulate bone remodeling either via exerting effects on osteoblasts and osteoclasts, through autocrine and paracrine mechanisms, or in the way of sensory nerve innervation. This suggests that Sema3A take a part in the skeletal biology. Accordingly, Sema3A is a promising new therapeutic agent for bone diseases.

## Author Contributions

All authors designed, wrote, checked, revised and finalized the manuscript.

## Funding

This work is supported by grants from the National Natural Science Foundation of China grant No. 81472078, 31470904, 31370992, 31670951 and Science and Technology Fund of Sichuan Province No. 2013SZ0057, Shenzhen basic research grant No. JCYJ20140417113430707, JSGG20160429190521240, JCYJ20140417113430708, JSGG20160429190521240; Shenzhen Peacock Plan KQCX2015033117354153.

## Conflict of Interest Statement

The authors declare that the research was conducted in the absence of any commercial or financial relationships that could be construed as a potential conflict of interest.

## References

[B1] Antipenko A. Himanen J. P. van Leyen K. Nardi-Dei V. Lesniak J. Barton W. A.. (2003). Structure of the semaphorin-3A receptor binding module. Neuron 39, 589–598. 10.1016/s0896-6273(03)00502-6 12925274

[B2] Behar O. Golden J. A. Mashimo H. Schoen F. J. Fishman M. C. (1996). Semaphorin III is needed for normal patterning and growth of nerves, bones and heart. Nature 383, 525–528. 10.1038/383525a0 8849723

[B3] Boyle W. J. Simonet W. S. Lacey D. L. (2003). Osteoclast differentiation and activation. Nature 423, 337–342. 10.1038/nature01658 12748652

[B4] Calvo W. Forteza-Vila J. (1969). On the development of bone marrow innervation in new-born rats as studied with silver impregnation and electron microscopy. Am. J. Anat. 126, 355–371. 10.1002/aja.1001260308 4188543

[B5] Carrer A. Moimas S. Zacchigna S. Pattarini L. Zentilin L. Ruozi G.. (2012). Neuropilin-1 identifies a subset of bone marrow Gr1- monocytes that can induce tumor vessel normalization and inhibit tumor growth. Cancer Res. 72, 6371–6381. 10.1158/0008-5472.can-12-0762 23222303

[B6] Case N. Rubin J. (2010). β-catenin—a supporting role in the skeleton. J. Cell. Biochem. 110, 545–553. 10.1002/jcb.22574 20512915 PMC3750230

[B7] Cawthorn W. P. Bree A. J. Yao Y. Du B. Hemati N. Martinez-Santibañez G.. (2012). Wnt6, Wnt10a and Wnt10b inhibit adipogenesis and stimulate osteoblastogenesis through a β-catenin-dependent mechanism. Bone 50, 477–489. 10.1016/j.bone.2011.08.010 21872687 PMC3261372

[B8] Chen H. He Z. Bagri A. Tessier-Lavigne M. (1998). Semaphorin-neuropilin interactions underlying sympathetic axon responses to class III semaphorins. Neuron 21, 1283–1290. 10.1016/s0896-6273(00)80648-0 9883722

[B9] Edoff K. Hellman J. Persliden J. Hildebrand C. (1997). The developmental skeletal growth in the rat foot is reduced after denervation. Anat. Embryol. (Berl) 195, 531–538. 10.1007/s004290050073 9193728

[B10] Elefteriou F. Campbell P. Ma Y. (2014). Control of bone remodeling by the peripheral sympathetic nervous system. Calcif. Tissue Int. 94, 140–151. 10.1007/s00223-013-9752-4 23765388 PMC3883940

[B11] Fukuda T. Takeda S. Xu R. Ochi H. Sunamura S. Sato T.. (2013). Sema3A regulates bone-mass accrual through sensory innervations. Nature 497, 490–493. 10.1038/nature12115 23644455

[B12] Giger R. J. Urquhart E. R. Gillespie S. K. Levengood D. V. Ginty D. D. Kolodkin A. L. (1998). Neuropilin-2 is a receptor for semaphorin IV: insight into the structural basis of receptor function and specificity. Neuron 21, 1079–1092. 10.1016/s0896-6273(00)80625-x 9856463

[B13] Gomez C. Burt-Pichat B. Mallein-Gerin F. Merle B. Delmas P. D. Skerry T. M.. (2005). Expression of semaphorin-3A and its receptors in endochondral ossification: potential role in skeletal development and innervation. Dev. Dyn. 234, 393–403. 10.1002/dvdy.20512 16145665

[B14] Goodman C. S. Kolodkin A. L. Luo Y. Püschel A. W. Raper J. A. (1999). Unified nomenclature for the semaphorins/collapsins. Semaphorin nomenclature committee. Cell 97, 551–552. 10.1016/s0092-8674(00)80766-7 10367884

[B15] Grayson W. L. Bunnell B. A. Martin E. Frazier T. Hung B. P. Gimble J. M. (2015). Stromal cells and stem cells in clinical bone regeneration. Nat. Rev. Endocrinol. 11, 140–150. 10.1038/nrendo.2014.234 25560703 PMC4338988

[B16] Hayashi M. Nakashima T. Taniguchi M. Kodama T. Kumanogoh A. Takayanagi H. (2012). Osteoprotection by semaphorin 3A. Nature 485, 69–74. 10.1038/nature11000 22522930

[B17] He X. Kuo Y. C. Rosche T. J. Zhang X. (2013). Structural basis for autoinhibition of the guanine nucleotide exchange factor FARP2. Structure 21, 355–364. 10.1016/j.str.2013.01.001 23375260 PMC3595398

[B18] He Z. Tessier-Lavigne M. (1997). Neuropilin is a receptor for the axonal chemorepellent semaphorin III. Cell 90, 739–751. 10.1016/s0092-8674(00)80534-6 9288753

[B19] Hill E. L. Turner R. Elde R. (1991). Effects of neonatal sympathectomy and capsaicin treatment on bone remodeling in rats. Neuroscience 44, 747–755. 10.1016/0306-4522(91)90094-5 1721689

[B20] Ieda M. Kanazawa H. Kimura K. Hattori F. Ieda Y. Taniguchi M.. (2007). Sema3a maintains normal heart rhythm through sympathetic innervation patterning. Nat. Med. 13, 604–612. 10.1038/nm1570 17417650

[B21] Janssen B. J. Malinauskas T. Weir G. A. Cader M. Z. Siebold C. Jones E. Y. (2012). Neuropilins lock secreted semaphorins onto plexins in a ternary signaling complex. Nat. Struct. Mol. Biol. 19, 1293–1299. 10.1038/nsmb.2416 23104057 PMC3590443

[B22] Janssen B. J. Robinson R. A. Pérez-Brangulí F. Bell C. H. Mitchell K. J. Siebold C.. (2010). Structural basis of semaphorin-plexin signalling. Nature 467, 1118–1122. 10.1038/nature09468 20877282 PMC3587840

[B23] Karsenty G. Ferron M. (2012). The contribution of bone to whole-organism physiology. Nature 481, 314–320. 10.1038/nature10763 22258610 PMC9047059

[B24] Kitsukawa T. Shimizu M. Sanbo M. Hirata T. Taniguchi M. Bekku Y.. (1997). Neuropilin-semaphorin III/D-mediated chemorepulsive signals play a crucial role in peripheral nerve projection in mice. Neuron 19, 995–1005. 10.1016/s0896-6273(00)80392-x 9390514

[B25] Koga T. Inui M. Inoue K. Kim S. Suematsu A. Kobayashi E.. (2004). Costimulatory signals mediated by the ITAM motif cooperate with RANKL for bone homeostasis. Nature 428, 758–763. 10.1038/nature02444 15085135

[B26] Kolodkin A. L. Levengood D. V. Rowe E. G. Tai Y. T. Giger R. J. Ginty D. D. (1997). Neuropilin is a semaphorin III receptor. Cell 90, 753–762. 10.1016/S0092-8674(00)80535-8 9288754

[B27] Koshihara Y. Kodama S. Ishibashi H. Azuma Y. Ohta T. Karube S. (1999). Reversibility of alendronate-induced contraction in human osteoclast-like cells formed from bone marrow cells in culture. J. Bone Miner. Metab. 17, 98–107. 10.1007/s007740050071 10340636

[B28] Kruger R. P. Aurandt J. Guan K. L. (2005). Semaphorins command cells to move. Nat. Rev. Mol. Cell Biol. 6, 789–800. 10.1038/nrm1740 16314868

[B29] Lee C. C. Kreusch A. McMullan D. Ng K. Spraggon G. (2003). Crystal structure of the human neuropilin-1 b1 domain. Structure 11, 99–108. 10.1016/s0969-2126(02)00941-3 12517344

[B30] Li Y. Yang L. He S. Hu J. (2015). The effect of semaphorin 3A on fracture healing in osteoporotic rats. J. Orthop. Sci. 20, 1114–1121. 10.1007/s00776-015-0771-z 26362654

[B31] Luo Y. Raible D. Raper J. A. (1993). Collapsin: a protein in brain that induces the collapse and paralysis of neuronal growth cones. Cell 75, 217–227. 10.1016/0092-8674(93)80064-l 8402908

[B32] McKenna C. C. Ojeda A. F. Spurlin J. Kwiatkowski S. Lwigale P. Y. (2014). Sema3A maintains corneal avascularity during development by inhibiting Vegf induced angioblast migration. Dev. Biol. 391, 241–250. 10.1016/j.ydbio.2014.04.017 24809797 PMC4103428

[B33] Moretti S. Procopio A. Lazzarini R. Rippo M. R. Testa R. Marra M.. (2008). Semaphorin3A signaling controls Fas (CD95)-mediated apoptosis by promoting Fas translocation into lipid rafts. Blood 111, 2290–2299. 10.1182/blood-2007-06-096529 18056484

[B34] Nakamura F. Tanaka M. Takahashi T. Kalb R. G. Strittmatter S. M. (1998). Neuropilin-1 extracellular domains mediate semaphorin D/III-induced growth cone collapse. Neuron 21, 1093–1100. 10.1016/s0896-6273(00)80626-1 9856464

[B35] Negishi-Koga T. Takayanagi H. (2012). Bone cell communication factors and semaphorins. Bonekey Rep. 1:183. 10.1038/bonekey.2012.183 24171101 PMC3810552

[B36] Neufeld G. Kessler O. (2008). The semaphorins: versatile regulators of tumour progression and tumour angiogenesis. Nat. Rev. Cancer 8, 632–645. 10.1038/nrc2404 18580951

[B37] Nogi T. Yasui N. Mihara E. Matsunaga Y. Noda M. Yamashita N.. (2010). Structural basis for semaphorin signalling through the plexin receptor. Nature 467, 1123–1127. 10.1038/nature09473 20881961

[B38] Okada S. Nakamura M. Katoh H. Miyao T. Shimazaki T. Ishii K.. (2006). Conditional ablation of Stat3 or Socs3 discloses a dual role for reactive astrocytes after spinal cord injury. Nat. Med. 12, 829–834. 10.1038/nm1425 16783372

[B39] Okubo M. Kimura T. Fujita Y. Mochizuki S. Niki Y. Enomoto H.. (2011). Semaphorin 3A is expressed in human osteoarthritic cartilage and antagonizes vascular endothelial growth factor 165-promoted chondrocyte migration: an implication for chondrocyte cloning. Arthritis Rheum. 63, 3000–3009. 10.1002/art.30482 21953086

[B40] Polleux F. Morrow T. Ghosh A. (2000). Semaphorin 3A is a chemoattractant for cortical apical dendrites. Nature 404, 567–573. 10.1038/35007001 10766232

[B41] Rodda S. J. McMahon A. P. (2006). Distinct roles for Hedgehog and canonical Wnt signaling in specification, differentiation and maintenance of osteoblast progenitors. Development 133, 3231–3244. 10.1242/dev.02480 16854976

[B42] Sandhu H. S. Herskovits M. S. Singh I. J. (1987). Effect of surgical sympathectomy on bone remodeling at rat incisor and molar root sockets. Anat. Rec. 219, 32–38. 10.1002/ar.1092190107 3688459

[B43] Serre C. M. Farlay D. Delmas P. D. Chenu C. (1999). Evidence for a dense and intimate innervation of the bone tissue, including glutamate-containing fibers. Bone 25, 623–629. 10.1016/s8756-3282(99)00215-x 10593406

[B44] Simonet W. S. Lacey D. L. Dunstan C. R. Kelley M. Chang M. S. Lüthy R.. (1997). Osteoprotegerin: a novel secreted protein involved in the regulation of bone density. Cell 89, 309–319. 10.1016/S0092-8674(00)80209-3 9108485

[B45] Sims N. A. Martin T. J. (2014). Coupling the activities of bone formation and resorption: a multitude of signals within the basic multicellular unit. Bonekey Rep. 3:481. 10.1038/bonekey.2013.215 24466412 PMC3899560

[B46] Soker S. Takashima S. Miao H. Q. Neufeld G. Klagsbrun M. (1998). Neuropilin-1 is expressed by endothelial and tumor cells as an isoform-specific receptor for vascular endothelial growth factor. Cell 92, 735–745. 10.1016/s0092-8674(00)81402-6 9529250

[B47] Takagawa S. Nakamura F. Kumagai K. Nagashima Y. Goshima Y. Saito T. (2013). Decreased semaphorin3A expression correlates with disease activity and histological features of rheumatoid arthritis. BMC Musculoskelet. Disord. 14:40. 10.1186/1471-2474-14-40 23343469 PMC3558329

[B49] Takahashi T. Fournier A. Nakamura F. Wang L. H. Murakami Y. Kalb R. G.. (1999). Plexin-neuropilin-1 complexes form functional semaphorin-3A receptors. Cell 99, 59–69. 10.1016/s0092-8674(00)80062-8 10520994

[B48] Takahashi T. Strittmatter S. M. (2001). Plexina1 autoinhibition by the plexin sema domain. Neuron 29, 429–439. 10.1016/s0896-6273(01)00216-1 11239433

[B50] Takeda S. (2008). Central control of bone remodelling. J. Neuroendocrinol. 20, 802–807. 10.1111/j.1365-2826.2008.01732.x 18601702

[B51] Takeda S. Elefteriou F. Levasseur R. Liu X. Zhao L. Parker K. L.. (2002). Leptin regulates bone formation via the sympathetic nervous system. Cell 111, 305–317. 10.1016/s0092-8674(02)01049-8 12419242

[B52] Takegahara N. Takamatsu H. Toyofuku T. Tsujimura T. Okuno T. Yukawa K.. (2006). Plexin-A1 and its interaction with DAP12 in immune responses and bone homeostasis. Nat. Cell Biol. 8, 615–622. 10.1038/ncb1416 16715077

[B53] Tamagnone L. Artigiani S. Chen H. He Z. Ming G. I. Song H.. (1999). Plexins are a large family of receptors for transmembrane, secreted and GPI-anchored semaphorins in vertebrates. Cell 99, 71–80. 10.1016/s0092-8674(00)80063-x 10520995

[B54] Taniguchi M. Yuasa S. Fujisawa H. Naruse I. Saga S. Mishina M.. (1997). Disruption of semaphorin III/D gene causes severe abnormality in peripheral nerve projection. Neuron 19, 519–530. 10.1016/s0896-6273(00)80368-2 9331345

[B55] Teitelbaum S. L. (2000). Bone resorption by osteoclasts. Science 289, 1504–1508. 10.1126/science.289.5484.1504 10968780

[B56] Togari A. Mogi M. Arai M. Yamamoto S. Koshihara Y. (2000). Expression of mRNA for axon guidance molecules, such as semaphorin-III, netrins and neurotrophins, in human osteoblasts and osteoclasts. Brain Res. 878, 204–209. 10.1016/s0006-8993(00)02700-1 10996153

[B57] Tolofari S. K. Richardson S. M. Freemont A. J. Hoyland J. A. (2010). Expression of semaphorin 3A and its receptors in the human intervertebral disc: potential role in regulating neural ingrowth in the degenerate intervertebral disc. Arthritis Res. Ther. 12:R1. 10.1186/ar2898 20051117 PMC2875625

[B58] Toyofuku T. Yoshida J. Sugimoto T. Zhang H. Kumanogoh A. Hori M.. (2005). FARP2 triggers signals for Sema3A-mediated axonal repulsion. Nat. Neurosci. 8, 1712–1719. 10.1038/nn1596 16286926

[B59] Tran T. S. Kolodkin A. L. Bharadwaj R. (2007). Semaphorin regulation of cellular morphology. Annu. Rev. Cell Dev. Biol. 23, 263–292. 10.1146/annurev.cellbio.22.010605.093554 17539753

[B60] Vadasz Z. Haj T. Halasz K. Rosner I. Slobodin G. Attias D.. (2012). Semaphorin 3A is a marker for disease activity and a potential immunoregulator in systemic lupus erythematosus. Arthritis Res. Ther. 14:R146. 10.1186/ar3881 22697500 PMC3446531

[B61] Wu X. Tu X. Joeng K. S. Hilton M. J. Williams D. A. Long F. (2008). Rac1 activation controls nuclear localization of β-catenin during canonical Wnt signaling. Cell 133, 340–353. 10.1016/j.cell.2008.01.052 18423204 PMC2390926

[B62] Xie W. Strong J. A. Mao J. Zhang J. M. (2011). Highly localized interactions between sensory neurons and sprouting sympathetic fibers observed in a transgenic tyrosine hydroxylase reporter mouse. Mol. Pain 7:53. 10.1186/1744-8069-7-53 21794129 PMC3152901

[B63] Yamashita T. Takahashi N. Udagawa N. (2012). New roles of osteoblasts involved in osteoclast differentiation. World J. Orthop. 3, 175–181. 10.5312/wjo.v3.i11.175 23330072 PMC3547111

[B64] Zanata S. M. Hovatta I. Rohm B. Püschel A. W. (2002). Antagonistic effects of Rnd1 and RhoD GTPases regulate receptor activity in semaphorin 3A-induced cytoskeletal collapse. J. Neurosci. 22, 471–477. 11784792 10.1523/JNEUROSCI.22-02-00471.2002 PMC6758682

[B65] Zhu Y. Romero M. I. Ghosh P. Ye Z. Charnay P. Rushing E. J.. (2001). Ablation of NF1 function in neurons induces abnormal development of cerebral cortex and reactive gliosis in the brain. Genes Dev. 15, 859–876. 10.1101/gad.862101 11297510 PMC312666

